# Development and dosimetric verification of static SHArc: Step‐and‐shoot carbon ion arc therapy for LET_d_ escalation in pancreatic tumors

**DOI:** 10.1002/mp.70055

**Published:** 2025-10-08

**Authors:** Filipa Baltazar, Thomas Tessonnier, Stewart Mein, Jakob Liermann, Abdallah Qubala, Jürgen Debus, Andrea Mairani

**Affiliations:** ^1^ Heidelberg Ion‐Beam Therapy Center (HIT) Department of Radiation Oncology Heidelberg Germany; ^2^ Clinical Cooperation Unit Radiation Oncology German Cancer Research Center (DKFZ) Heidelberg Germany; ^3^ Medical Faculty Heidelberg University Heidelberg Germany; ^4^ Clinical Cooperation Unit Translational Radiation Oncology German Cancer Research Center (DKFZ) Heidelberg Germany; ^5^ Department of Accelerator and Medical Physics Institute for Quantum Medical Science National Institutes for Quantum Science and Technology Chiba Japan; ^6^ Heidelberg Institute of Radiation Oncology (HIRO) Heidelberg Germany; ^7^ Department of Radiation Oncology Heidelberg University Hospital Heidelberg Germany; ^8^ National Center for Tumor diseases (NCT) Heidelberg Germany; ^9^ German Cancer Consortium (DKTK) Core Centre Heidelberg Heidelberg Germany; ^10^ Centro Nazionale di Adroterapia Oncologica (CNAO) Pavia Italy

**Keywords:** heavy‐ion gantry, LET_d_‐boosting, pancreatic cancer, spot‐scanning hadron arc, static arc

## Abstract

**Background:**

Carbon ion radiotherapy (CIRT) offers higher linear energy transfer (LET) and superior relative biological effectiveness, making it a promising option for treating hypoxic, radioresistant tumors. Spot‐scanning Hadron Arc (SHArc) therapy enables dose‐averaged LET (LET_d_) escalation in the tumor but increases planning and setup complexity. Dynamic delivery remains impractical for gantry‐based carbon ion arc therapy due to the system's large size and complex control requirements. Step‐and‐shoot delivery, while less efficient, provides a feasible alternative and represents a key step towards clinical SHArc implementation.

**Purpose:**

This work establishes the first step‐and‐shoot planning and delivery technique for carbon ion arc therapy (static SHArc) at the Heidelberg Ion‐beam Therapy Center using the gantry system. Static SHArc therapy is evaluated in terms of plan quality and delivery feasibility for pancreatic cancer.

**Methods:**

Static SHArc plans were optimized for seven pancreatic cancer cases, considering 20 gantry angles. Two distinct energy layer (EL) selection techniques were investigated: (1) Central EL, selecting the seven central ELs per beam, and (2) MU‐Based EL, prioritizing ELs contributing the highest monitor units (MU). LET_d_ optimization was performed to escalate the minimum LET_d_ to ∼50–80 keV/µm within the gross tumor volume. Static SHArc plans were compared against conventional IMPT using two single‐field optimized posterior oblique beams (2‐SFO), in terms of dose conformality, LET_d_, and robustness against setup (5 mm) and range (1.5%) uncertainties. Inter‐fractional robustness was assessed via forward dose calculation on daily control CT scans. Dosimetric validation and delivery time verification for static SHArc using the heavy ion gantry system were conducted via end‐to‐end testing with ion chamber and film measurements in a cylindrical PMMA phantom.

**Results:**

Static SHArc plans improved LET_d_ distributions in the tumor without compromising target coverage and clinical OAR constraints. The MU‐based EL method increased minimum target dose, whereas Central EL enabled higher LET_d_ concentration in the tumor center. Both energy selection methods for static SHArc exhibited reduced inter‐fractional robustness compared to the two‐SFO. Dosimetric verification showed deviations < 3% and total delivery time was ∼27 min.

**Conclusions:**

This study investigates static SHArc, a step‐and‐shoot approach for delivering carbon ion arc therapy. While static SHArc can provide dosimetric advantages, particularly in terms of LET_d_ distribution, EL selection plays a key role. Improving inter‐fraction robustness remains crucial for clinical implementation.

## 1 INTRODUCTION

Carbon ion radiotherapy (CIRT) offers superior relative biological effectiveness (RBE) and higher linear energy transfer (LET). Higher LET reduces the oxygen enhancement ratio (OER), making CIRT less sensitive to tumor oxygenation changes.^1^ As a result, CIRT may be more effective against hypoxia‐induced radioresistant tumors, a challenge that conventional photon‐based radiotherapy (RT) still faces.^2^

In recent years, proton arc therapy (PAT) has emerged as an advanced intensity‐modulated proton therapy (IMPT) technique that enhances precision by rotating the beam around the patient for an increased degree of freedom in treatment optimization. PAT can be delivered using two different modes: dynamic (continuous rotation) and step‐and‐shoot (static). Studies have highlighted PAT's dosimetric advantages over standard IMPT in protons across various clinical sites, including oropharyngeal,^3^ brain,^4^, ^5^, ^6^ breast^7^ and lung^8^, ^9^, ^10^ cancers. PAT using heavier ions like helium, carbon, oxygen, and neon, known as Spot‐scanning Hadron Arc (SHArc) therapy, has been proposed^11^ with research by Mein et al. demonstrating SHArc's potential to enhance dose‐averaged linear energy transfer (LET_d_) within the gross tumor volume (GTV).^12^, ^13^ Recently, Tessonnier et al. conducted the first SHArc delivery and dosimetric validation using carbon ions in a phantom, providing experimental evidence of SHArc's potential to overcome hypoxia‐induced radioresistance.^14^

Key milestones were recently made in developing a gantry‐based PAT system for robust and efficient dynamic PAT.^15^ However, no clinical delivery system currently exists for carbon ion arc therapy using either dynamic or step‐and‐shoot. Furthermore, dynamic delivery remains a major challenge for gantry‐based carbon ion arc therapy and is presently considered infeasible due to the immense gantry size (> 600t) and the complexities of control system design. Step‐and‐shoot delivery, while potentially less efficient than dynamic delivery, is therefore an important next stage of PAT development and should serve as an interim solution for gantry‐based SHArc therapy.

Although technically feasible, works have yet to establish and demonstrate robust and deliverable methods for step‐and‐shoot SHArc. There are several challenges that must be addressed due to the added complexity in the treatment planning and delivery process. For example, the increased number of beams and prolonged energy layer (EL) switching times using single energy extraction (SEE) synchrotron‐based scanning systems may significantly increase both optimization and irradiation times if not explicitly considered during plan optimization. Various energy selection strategies have been proposed in the literature to address these challenges. Examples of such strategies in proton therapy include mono‐energetic beam selection^16^ and the Spot‐scanning Proton Arc (SPArc) algorithm, which takes an iterative approach to integrate plan robustness into energy selection but is computationally intensive.^17^ Recently, Wuyckens et al. proposed a hybrid approach combining geometry‐based EL pre‐selection with dose‐based filtering, including the so‐called “unrestricted filtering,” which accounts for EL monitor units (MU) during selection.^18^ Another approach, suggested by Engwall et al., involves dividing static arc plans into subplans, which are then delivered across multiple fractions throughout the treatment course.^19^ For SHArc, most studies conducted to date have used beams with single ELs selected based on Bragg Peaks terminating near the tumor's center to boost LET_d_. A recent work by Volz et al. investigated additional methods for EL selection, including a sophisticated filtering approach that incorporates dose distribution into the EL selection.^20^

In this work, a dedicated treatment planning and delivery technique for step‐and‐shoot carbon ion arc therapy (static SHArc) is developed using the heavy ion gantry system at the Heidelberg ion‐beam therapy center (HIT). Pancreatic cancers are often hypoxic and radioresistant, with previous studies showing a correlation between the minimum LET_d_ in the GTV and improved local control.^21^ Therefore, dedicated LET_d_ escalation strategies like SHArc are needed to improve clinical outcomes. This study provides the first investigation of static SHArc therapy for pancreatic cancer, assessing its dosimetric benefits and clinical feasibility within current delivery constraints, while highlighting key challenges for future clinical implementation.

## 2 METHODS

### 2.1 Patient data

This study analyzed a cohort of seven patients with locally advanced pancreatic cancer (LAPC) treated at the Heidelberg ion‐beam therapy center (HIT) within the PACK trial.^22^ In this clinical trial, patients were treated with carbon ions, using two posterior gantry fields (160° and 200°), which were optimized as single field optimized (SFO) plans. To account for target and organ motion during treatment, Four‐dimensional computed tomography (4DCT) was performed for individualized margin expansion of the clinical target volume (CTV) to the internal target volume (ITV) and identification of gastrointestinal (GI) movement. The patients selected for the current analysis present GTV ranging from 7.20 to 92.55 cc.

### 2.2 Treatment planning strategies

Static SHArc plans were generated using the 20 clinically available beam angles for carbon ions at HIT (Figure A1(a) in Supplementary Material). For comparison purposes, treatment plans using two posterior beams were also created for each patient, following the clinical standard approach employed in the PACK trial (referred to as 2‐SFO). All plans were optimized using RayStation 2024A‐DTK treatment planning system (TPS) (Raysearch Laboratories, AB), with a prescribed dose (D_pres_) of 48 Gy(RBE) delivered to the CTV across 12 fractions. The RBE‐weighted dose was calculated using the local effect model (LEM I)^23^ considering an α/β ratio of 5 Gy for the ITV and 2 Gy for surrounding tissues, under HIT's clinical standards for the PACK cohort.

Dose objectives for plan optimization are summarized in Table A1 (Supplementary Material). Although plans were optimized to aim for 90% of the CTV to receive 90% of the prescribed dose with homogenous dose coverage, priority was given to limiting the maximum dose to the GI tract. When the constraints for the GI tract could not be met, the CTV coverage was reduced. The GTV minimum and maximum doses, as well as the maximum dose to the GI tract, were robustly optimized with ± 7 mm setup shifts, evaluating 15 scenarios for each optimization iteration. LET_d_ optimization feature in RayStation was used for plan optimization, setting minimum LET_d_ objectives of 50 to 80 keV/µm, based on GTV size. Lower thresholds were applied for larger volumes, where LET_d_ boosting is more challenging, to ensure robust feasibility while remaining above the 44 keV/µm reported by Hagiwara et al. for improved local control in pancreatic cancer.

The beam specifications included a minimum beam width in air at the isocenter of 10 mm, following a hexagonal spot pattern with a 3.6 mm spot spacing, and a 3.1 mm EL spacing. The optimization considered an intensity‐controlled raster scanning system and a step‐and‐shoot approach for SHArc delivery.

#### 2.2.1 Energy layer selection strategies

To balance treatment delivery time and plan quality, both in terms of tumor coverage and robustness, two energy layer filtering approaches were used:

1. **Central EL**: In line with previous SHArc studies,^14^, ^24^ this method retains ELs whose Bragg peaks terminate near the center of the target. To balance delivery time and plan robustness, the 7 central ELs were kept for each beam (resulting in a total of 140 EL per plan). For this, beam spots were initially positioned based on beam specifications and tumor geometry, and later filtered to retain the 7 ELs with Bragg peaks closest to the tumor center, thereby focusing high‐LET_d_ within the tumor core.
2. **MU‐based EL**: Inspired by the work of Wuyckens et al.^18^ this method retains the ELs contributing the most MUs. An initial optimization using all ELs was first performed, applying relevant dose‐ and LET_d_‐based objectives for the intended plan. The ELs were then filtered based on their MU contribution, and beams with no remaining ELs after filtering were excluded. A second optimization was subsequently carried out to ensure that all planning objectives were met with the reduced number of ELs. The percentage of ELs retained for each plan was chosen to ensure irradiation times were comparable to those achieved with the Central EL approach.

Figure A1(b) (in Supplementary Material) illustrates their differences in terms of EL selection for an example case.

### 2.3 Plan evaluation

Target coverage was assessed by examining the dose received by at least 90% of the target volume (D_90%_), the homogeneity index (HI = (D_2%_—D_98%_) / D_50%_), and the percentage of the target volume receiving at least 95% of the prescribed dose (V_95%_). In terms of LET_d_, the minimum and maximum values in the GTV were assessed as LET_d98%_ and LET_d1%_, respectively, along with the maximum LET_d_ in the GI tract, LET_d1%_. Normal tissue complication probability (NTCP) for different toxicity endpoints in the GI tract was evaluated based on Lyman–Kutcher–Burman (LKB), as described in detail in the Supplementary Material (B). Additionally, pairwise two‐sided Wilcoxon signed‐rank tests of relevant dose and LET_d_ metrics were performed using SciPy, with p‐values below 0.05 considered statistically significant. Further details are provided in Supplementary Material (C).

#### 2.3.1 Robust evaluation

The planning strategies were compared through a robust evaluation, on the planning CT, with isocenter shifts and range uncertainty assessed separately. For setup uncertainty, 14 isocenter shifts of up to 5 mm were analyzed‐ 6 along the main axes and 8 along the diagonals. Two density variation scenarios (± 1.5%) were also evaluated. Robust target coverage was assessed using two criteria: 1) GTV: D_95% _> 95% D_pres_; 2) GTV: D_1% _< 105% D_pres_. For OAR robustness, the focus was on the maximum dose to the GI tract, particularly D_1cc_ ≤ 43.2 Gy(RBE).

#### 2.3.2 Inter‐fractional variation

Two additional 3DCT scans (control‐1 and control‐2) were acquired for each patient during treatment, 1 week apart. These scans were registered to the initial planning CT, and target volumes were re‐delineated. To assess the impact of inter‐fraction variations, the original treatment plans were recalculated on the new images, with tumor coverage evaluated based on dose distribution and LET_d_. In these recalculations, no additional positioning or density uncertainties were considered for the control CTs.

#### 2.3.3 Treatment delivery time

The delivery times were estimated considering the constraints of our heavy‐ion gantry system at HIT and employing a step‐and‐shoot approach. The assumptions for these calculations included a gantry rotation speed of 3°/s, a spot irradiation time of 2 ms, and an EL switching time of 5 s. An additional 45 s were added to account for processing between beams. The delivery times for a multi‐energy mode (MEM) scenario were also computed. In MEM, particles are accelerated and switched between different ELs until the total particle count per spill is reached (4 × 10^8^ particles for the case of carbon ions), after which the particles are reinjected into the synchrotron. This approach can reduce irradiation time, with each acceleration within the spill being 0.2 s compared to the 4 s needed for switching ELs in conventional methods.

### 2.4 Dosimetric verification of static SHArc plans at the HIT gantry treatment room

**FIGURE 1:**
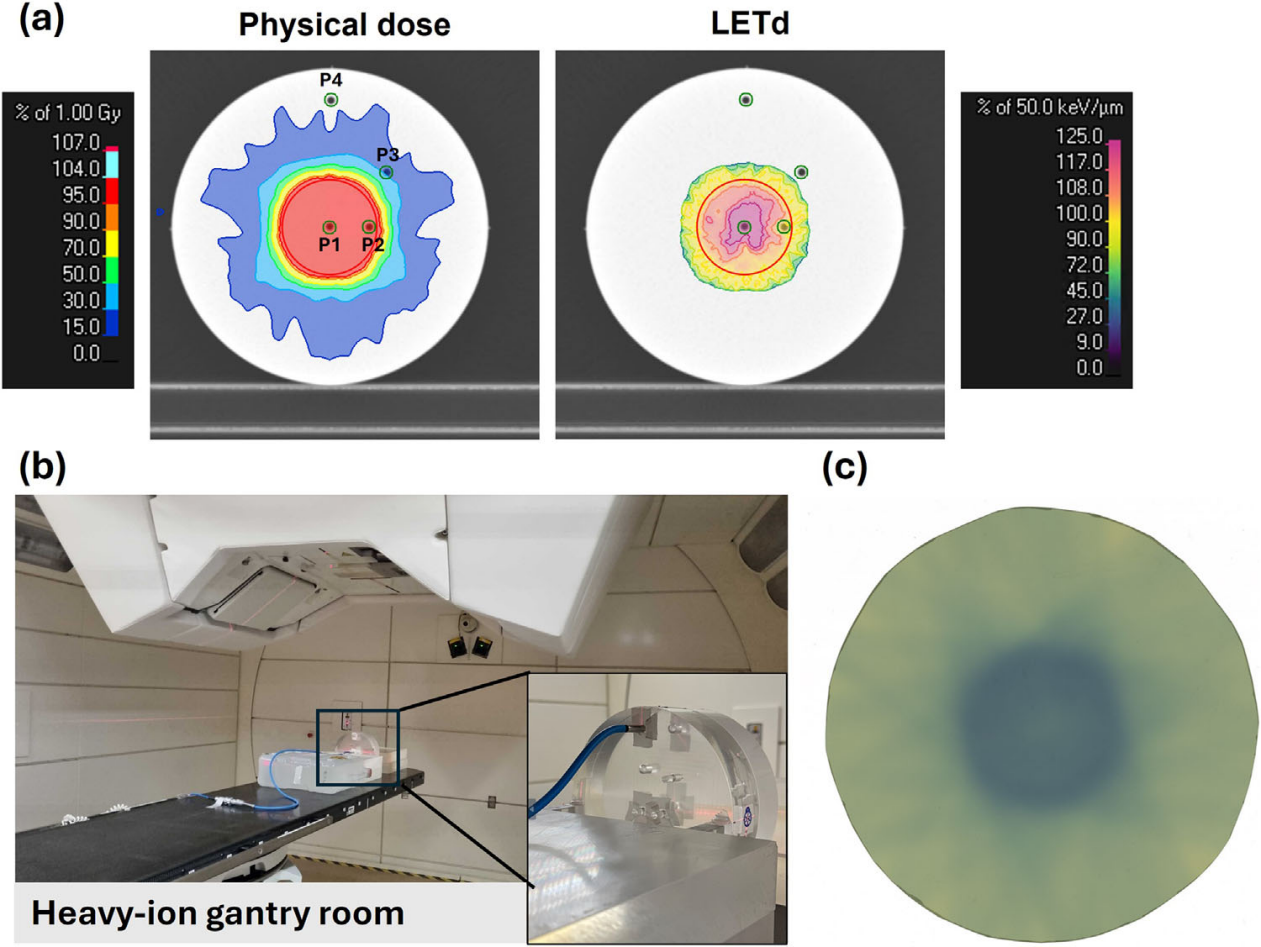
(a) Physical and LET_d_ distributions of the plan delivered at the gantry treatment room, with the CTV outlined in red and measurement points (P1–P4) marked in green. For the LET_d_ distribution, a 1.25 Gy(RBE) dose threshold was applied, corresponding to approximately 50% of the CTV's RBE‐weighted dose, as calculated using the LEM‐I model with α/β = 5 Gy. (b) Experimental setup used for irradiation, with a zoomed‐in view showing the PMMA cylindrical phantom, where the pinpoint chamber is inserted at P4. (c) Radiochromic film used for qualitative analysis, irradiated with a scaled version of the 1 Gy plan to 5 Gy. CTV, clinical target volume; LEM, local effect model; LET_d_, dose‐averaged linear energy transfer; RBE, relative biological effectiveness.

Extending on previous work by Tessonnier et al.^14^ a dosimetric verification of a static SHArc plan was conducted at the HIT gantry, using a cylindrical polymethyl methacrylate phantom, which includes four inserts for ionization chamber placement (PinPoint‐TM31015, PTW). Treatment plans were created in RayStation 2024A‐DTK (RaySearch Laboratories) for a cylindrical shaped CTV (3 cm radius, 3.5 cm height). A static SHArc plan was optimized to ensure a uniform, conformal 1 Gy physical dose to the target. Consistent with the in‐silico analysis presented in this work, the plan initially included the 20 clinically commissioned beam angles at HIT. The MU‐based filtering method was utilized for EL selection to improve the robustness of the performed measurements. The total number of ELs was constrained to approximately 140 to align with the rest of the study, resulting in 18 beams being used for irradiation after filtering. LET_d_ optimization was used to concentrate high LET_d_ within the CTV while limiting its maximum values outside the target. This was achieved by setting a minimum LET_d_ objective of 50 keV/µm for the CTV and 60 keV/µm for a smaller GTV, defined by contracting the cylinder by 2 cm in radius and 1 cm in height. The maximum LET_d_ outside the tumor was also restricted during optimization. The resulting physical dose and LET_d_ distributions of the irradiated plan are displayed in Figure 1a. The experimental set‐up is shown in Figure 1b.

The plan was measured at the four positions (P1–P4) following clinical protocols.^25^ Additionally, a radiochromic film (Gafchromic EBT, Advanced Materials Group, International Specialty Products) was irradiated with a scaled 5 Gy plan for qualitative verification, shown in Figure 1c. During the first measurement at each point, the phantom's position was continuously monitored using the optical surface guided AlignRT system^26^ (version 5.1.2, VisionRT Ltd, London, United Kingdom).

## 3 RESULTS

### 3.1 Plan evaluation: RBE‐dose distribution

**FIGURE 2:**
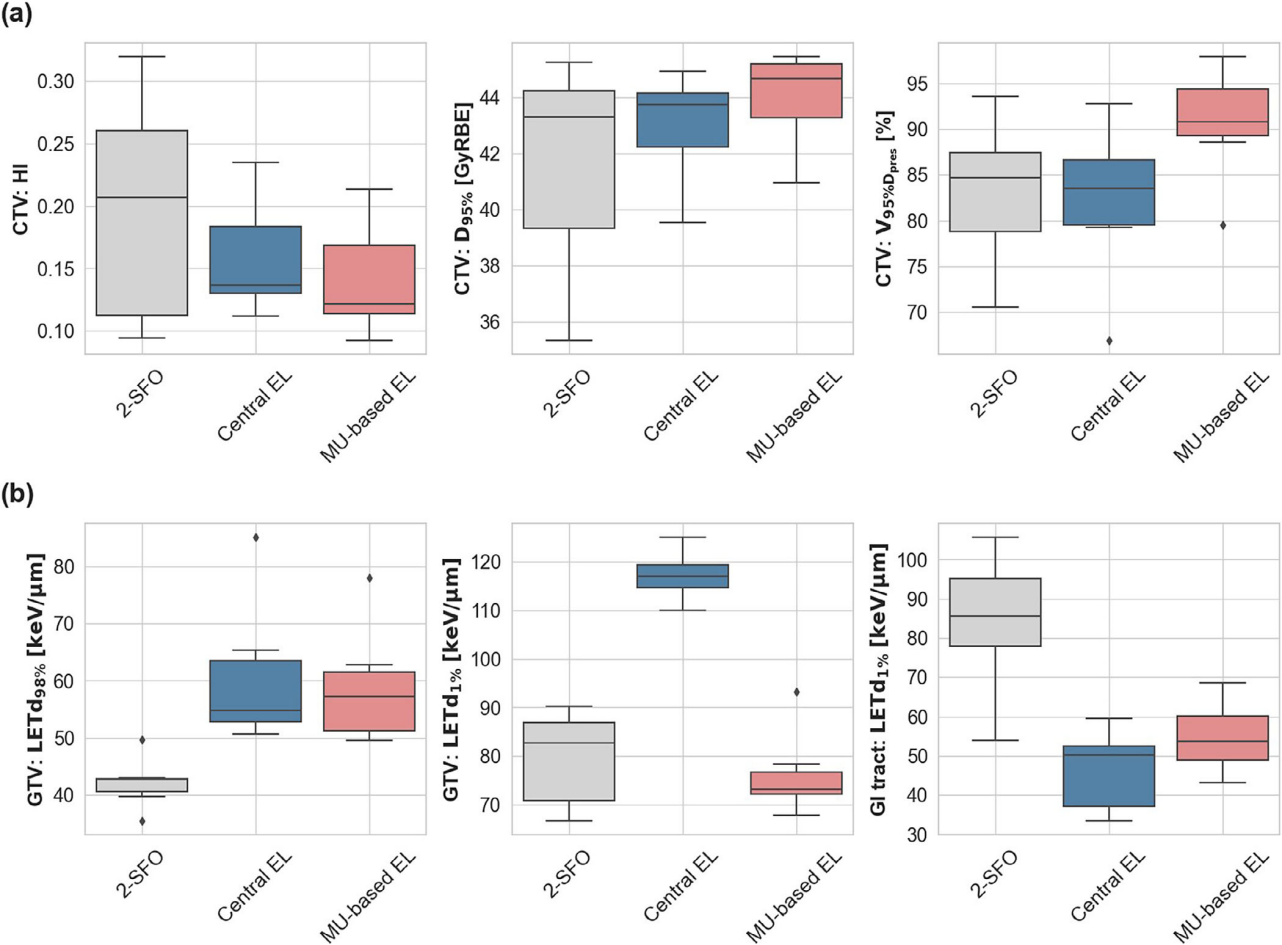
Distribution of (a) dose metrics—HI, D_95%_, and V_95%_ in the CTV—and (b) LET_d_ metrics—LET_d98%_ and LET_d1%_ in the GTV, as well as LET_d1%_ in the gastrointestinal tract (GI tract) for the three optimization strategies across the 7 patient‐cohort. CTV, clinical target volume; GTV, gross tumor volume; HI, Homogeneity Index; LET_d_, dose‐averaged linear energy transfer.

Table 1 summarizes the dosimetric outcomes for the three planning strategies: 2‐SFO (the clinical standard at HIT for PACK patients), Central EL, and MU‐based EL. An asterisk (*) marks metrics where the specific static SHArc strategy showed a statistically significant difference compared to 2‐SFO. A more detailed statistical analysis is provided Supplementary Material (C). Additional CTV‐specific dosimetric parameters are illustrated in Figure 2a, with patient‐specific DVH plots provided in the Supplementary Material (D). All plans adhered to maximum dose constraints for OAR. Overall, both static SHArc strategies were able to match the target coverage achieved by the 2‐SFO plans, with an increase in the mean D_95%_ to the CTV being 43.01 Gy(RBE) for Central EL and 43.96 Gy(RBE) for MU‐based EL, compared to 41.57 Gy(RBE) for the 2‐SFO plan. However, both static SHArc plans resulted in an increase in low‐dose bath, as indicated by the significant increase in the percentage of body volume receiving at least 10% of the prescribed dose (V_4.8_ _Gy(RBE)_). Specifically, the mean V_4.8_ _Gy(RBE)_ was 9.86% for 2‐SFO, which increased to an approximate median value of 21% for both static SHArc EL filtering methods.

**TABLE 1:** Dose metrics (mean [minimum‐maximum] values) within the 7 patient‐cohort for target coverage (CTV) and organs at risk (OAR) dose constraints across the three optimized plans for each patient: 2‐SFO, static SHArc: Central EL, and static SHArc: MU‐based EL.

	Planning strategy		
ROI: Dose metric	2‐SFO mean (min.–max.)	Central EL mean (min.–max.)	MU‐based EL mean (min.–max.)
CTV: D_95%_ (Gy(RBE))	41.57 (35.32–45.25)	43.0 (39.54–44.93)	44.0 (40.94–45.44)*
CTV: D_90%_ (Gy(RBE))	43.41 (37.39–46.28)	44.41 (41.56–46.13)	45.48 (43.0–46.4)*
CTV: V_95%Dpres_ (%)	83.03 (70.56–93.52)	82.21 (66.9–92.74)	90.77 (79.53–97.87)*
CTV: HI	0.2 (0.09–0.32)	0.16 (0.11–0.24)	0.14 (0.09–0.21)
GI tract: D_max_ (Gy(RBE))	43.15 (40.28–46.04)	44.08 (42.91–45.2)	44.29 (42.89–45.52)
GI tract‐ITV: D_max_ (Gy(RBE))	42.9 (40.27–46.04)	43.09 (42.91–43.19)	43.05 (42.89–43.2)
Spinal Cord D_max_ (Gy(RBE))	32.48 (23.06–34.8)	17.49 (12.98–22.83)*	19.25 (13.27–25.91)*
Kidney (right): V_24Gy(RBE)_ (%)	6.13 (0.0–20.2)	3.25 (0.0–9.18)	3.08 (0.0–12.08)
Kidney (left): V_24Gy(RBE)_ (%)	5.06 (0.01–12.05)	0.86 (0.0–4.3)	1.34 (0.0–7.24)
Body: V_4.8_ _Gy(RBE)_ (%)	9.86 (8.07–13.74)	21.47 (11.82–40.39)*	20.98 (12.04–40.68)*

*Note*: Metrics with an asterisk (*) indicate statistically significant differences between the corresponding SHArc plan and the 2‐SFO reference.

Abbreviations: EL, energy layer; MU, monitor units; SHArc, Spot‐scanning Hadron Arc.

Among SHArc strategies, the MU‐based EL approach offered better flexibility in achieving more homogeneous and conformal CTV coverage compared to Central EL, while maintaining OAR constraints. This is reflected in HI and V_95%_, with MU‐based EL showing mean values of 0.14 [0.09–0.21] for HI and 90.77% [79.53–97.87] for V_95%_, compared to Central EL's 0.16 [0.11–0.24] and 82.21% [66.9–92.74], respectively.

The NTCP calculations, performed for three endpoints, showed no significant differences between the static SHArc approaches and 2‐SFO. The largest mean difference was observed for the diarrhea endpoint, with increases of approximately 0.4% for both SHArc strategies, as detailed in Figure B1 of the Supplementary Material (B).

#### 3.1.1 Plan evaluation: LETd distribution

Figure 2b illustrates the distribution of minimum and maximum LET_d_ in the GTV and maximum LET_d_ in the GI tract for the optimized plans across the cohort. In general, static SHArc plans increased the minimum LET_d_ in the GTV while reducing the maximum LET_d_ in the GI tract compared to 2‐SFO. Specifically, the cohort's median LET_d98%_ in the GTV increased from 42.6 keV/µm in 2‐SFO to 54.8 and 57.2 keV/µm for Central EL and MU‐based EL, respectively. Simultaneously, the maximum LET_d_ in the GI tract (LET_d1%_) decreased from a median value of 85.5 keV/µm in 2‐SFO to 50.2 and 53.6 keV/µm with the two static SHArc approaches. Among the SHArc strategies, the Central EL approach concentrated the high‐LET_d_ within the tumor center, often considered the hypoxic core, as translated by the higher GTV LET_d1%_ values. Specifically, the Central EL optimized plan led to an approximate median of 117.0 keV/µm [110.0‐124.9], compared to 73.26 keV/µm [67.8‐93.3] for the MU‐based EL. An axial slice of the LET_d_ distribution for one patient, reflecting the high‐LET_d_ region in the tumor center with the Central EL approach, compared to the MU‐based EL approach, is shown in the Supplementary Material (E).

### 3.2 Robust evaluation

**FIGURE 3:**
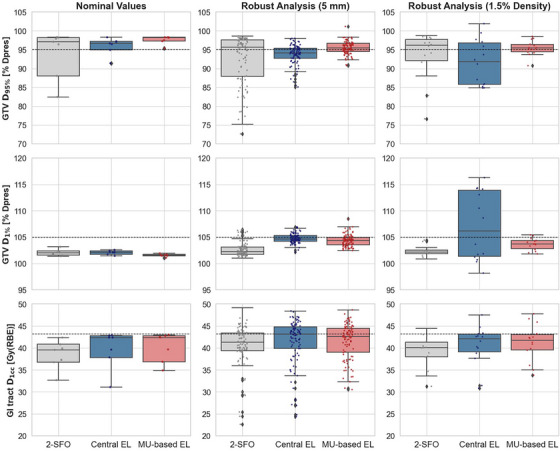
Robust evaluation of GTV coverage (D_95%_ and D_1%_) and maximum GI tract dose (D_1cc_) under positional (5 mm) and range (1.5%) uncertainties. Rows correspond to the evaluated clinical goals: (1) GTV D_95%_ > 95% of the prescribed dose (D_pres_), (2) GTV D_1%_ < 105% of the D_pres_, and (3) GI tract D_1cc_ < 43.2 Gy(RBE). Each boxplot shows the dose distribution for either nominal case or robustness scenarios, for one of the evaluated plans: two‐SFO (gray), Central EL (blue), and MU‐based EL (red). The nominal case includes 7 values, while the robustness analysis consists of 98 positional uncertainty results (14 scenarios × 7 patients) and 14 range uncertainty results (2 scenarios × 7 patients). The scattered points consist of the different dose metrics obtained from each robust evaluation scenario. GTV, gross tumor volume; GI, gastrointestinal; RBE, relative biological effectiveness.

Figure 3 presents the robust analysis of the three planning strategies under positional (5 mm, 14 scenarios) and density (1.5%, 2 scenarios) uncertainties, evaluating GTV coverage (D_95%_ and D_1%_, as percentage of prescribed dose) and maximum GI tract dose (Gy(RBE)). The 2‐SFO plan shows greater variation in D_95%_, ranging from 72.63 to 98.65% for positional uncertainties and 76.65 to 98.67% for density perturbations. Among static SHArc plans, the Central EL strategy appears less robust, especially under density perturbations, where the robust evaluation results in a wider range of D_95%_. A similar behavior is seen for GTV D_1%_: under positional uncertainties, both static SHArc plans maintain a median D_1%_ below 105% of the prescribed dose, with the MU‐based EL generally yielding lower values than Central EL. Under density variations, however, the Central EL plan reaches a higher median D_1%_ of ∼106%, ranging from 98.17 to 116.27 %. For GI tract dose, the median D_1cc_ remains below the evaluation criteria across all plans, for position and density robustness evaluation. Although maximum GI tract D_1cc_ under positional uncertainties is similar across planning strategies (∼49 Gy(RBE)), density perturbations result in higher doses in both static SHArc plans compared to SFO (approximately 47.5 Gy(RBE) compared to 44.5 Gy(RBE)).

### 3.3 Inter‐fractional variation

**FIGURE 4:**
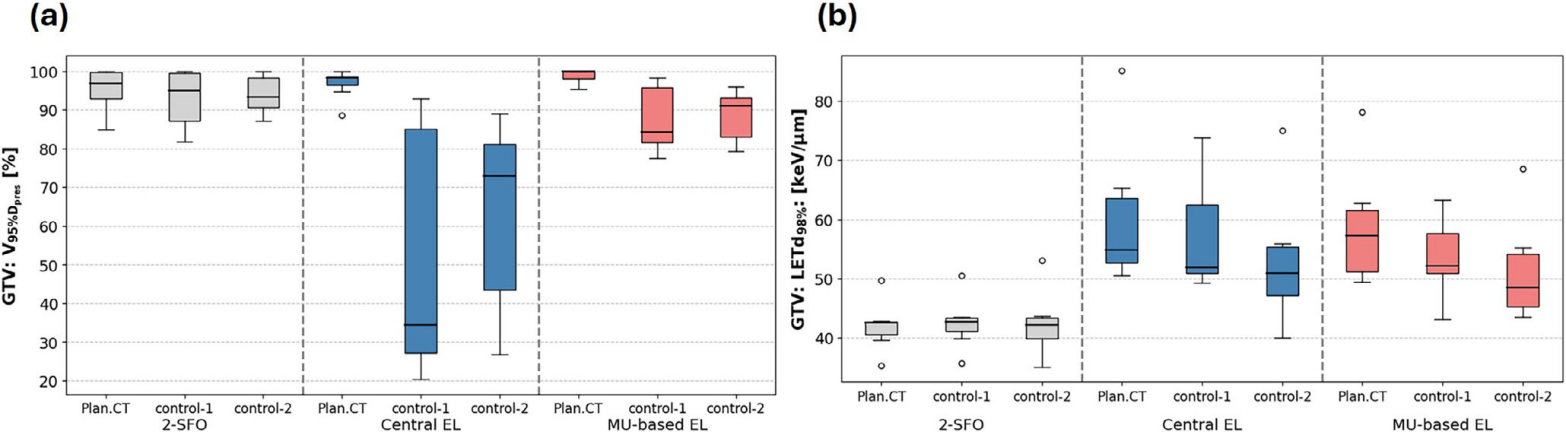
Distribution of (a) minimum GTV coverage (V_95%_) and (b) minimum LET_d_ (LET_d98%_) for the initial plan (Plan. CT) and recalculations on control CTs 1 and 2 across seven patients, comparing 2‐SFO (gray), Central EL (blue), and MU‐based EL (red) planning strategies. EL, energy layer; GTV, gross tumor volume; LET_d_, dose‐averaged linear energy transfer.

Static SHArc plans exhibited greater susceptibility to inter‐fractional changes, impacting both target coverage (V_95%_) and minimum LET_d_ (LET_d98%_), as summarized in Figure 4. The Central EL strategy showed the most pronounced V_95%_ reduction, with median values decreasing from 98.25% [88.67–99.84] in the planning CT to 34.36% [20.5–93.01] and 72.98% [26.79–89.02] in control CTs 1 and 2, respectively. A case with substantial tumor coverage loss between the planning CT and control CT 1, highlighting the impact of anatomical changes on dose distribution, is illustrated in the Supplementary Material (F). In terms of LET_d_ distribution, while LET_d98%_ was also impacted, it remained above 50 keV/µm for both static SHArc strategies, exceeding the 2‐SFO plan (∼42 keV/µm).

### 3.4 Treatment delivery time

The 2‐SFO plans require around 7 min for irradiation, with the total treatment times typically reaching about 10 min. The optimized static SHArc plans included 16 to 20 beams, with 127 to 140 ELs in total. Based on these parameters and using current gantry settings at HIT, the estimated delivery time for static SHArc plans in conventional mode ranges from 25 to 28 min. In addition, it was estimated that delivery times could potentially be reduced to 16 to 20 min if MEM would be considered.

### 3.5 Dosimetric verification at the HIT gantry treatment room

Table 2 summarizes the measurements conducted at the HIT gantry, detailing the LET_d_ values computed in RayStation for each point (P1‐P4). Following clinical quality assurance protocols^25^ the deviation to the TPS dose was computed considering the measured‐to‐calculated dose difference (D_meas_‐D_TPS_), normalized to the maximum dose calculated in the plan (D_max_). As shown in the table, dose deviations from the TPS were the largest at the central position (P1, < 2.8%) while mean variations at the other positions were < 0.9%. The delivery time for the irradiated plan was approximately 27 min.

**TABLE 2:** Summary of dosimetric measurements performed at the heavy‐ion gantry at HIT, including the corresponding LET_d_ values for each measurement point (P1–P4).

Plan details	Position	LET_d_ (keV/µm)	Deviation to TPS (mean ± std)
SHArc plan with 18 beams, using MU‐based EL selection Plan optimized for 1 Gy	P1	67.3	−2.75% ± 0.22%
	P2	51.2	−0.38% ± 0.16%
	P3	20.5	+0.86% ± 0.27%
	P4	14.7	+0.22% ± 0.07%

*Note*: The deviation from TPS calculations is reported following clinical QA procedures. For each measurement point, the table presents the mean value from repeated measurements (three times) along with the corresponding standard deviation. The total number of ELs of the static SHArc plan irradiated was filtered using the MU‐based EL strategy.

Abbreviations: EL, energy layer; HIT, Heidelberg ion‐beam therapy; LET_d_, dose‐averaged linear energy transfer; MU, monitor units; SHArc, Spot‐scanning Hadron Arc; TPS, treatment planning system.

## 4 DISCUSSION

In this work, the first dedicated treatment planning and optimization technique for static SHArc radiotherapy was developed and applied to escalate LET_d_ in pancreatic tumors. Plans were generated for seven patients previously treated at HIT in the PACK trial, using the clinical 2‐SFO beam arrangement and SHArc strategies. For the latter, a step‐and‐shoot approach (static SHArc), compatible with HIT's current carbon ion delivery capabilities, was employed with 20 gantry angles commissioned for carbon ions. To limit both optimization and irradiation for the SHArc plans, two EL reduction strategies were used: the Central EL method, focusing on layers with range peaks at the tumor center, and the MU‐based approach, prioritizing layers with higher MU contributions. SHArc plans were optimized considering robust optimization, explicitly accounting for setup uncertainties (total of 15 optimized scenarios), as well as LET_d_ optimization, aimed at increasing the minimum LET_d_ in the GTV (to a minimum of 50 kev/µm). SHArc plans were compared to the clinical 2‐SFO standard in terms of dose distribution, LET_d_, robustness to uncertainties, and inter‐fractional variations. Finally, to assess the feasibility of step‐and‐shoot SHArc delivery at the heavy‐ion gantry, an additional plan was created and irradiated, and dose measurements were performed for validation.

Both static SHArc strategies were able to match the dose coverage achieved with standard 2‐SFO plans, while increasing the minimum LETd within the GTV. Although an increase in the low‐dose bath was observed (expressed in terms of V_2.4_ _Gy(RBE)_/V_4.8_ _Gy(RBE)_), all SHArc plans were optimized to adhere to clinical dose constraints for the different OAR. Moreover, NTCP calculations for various endpoints, specifically gastrointestinal hemorrhage, obstruction/perforation, and diarrhea, suggested that the increase in the low‐dose bath in the GI tract for SHArc plans does not meaningfully affect NTCP predictions and, consequently, should not lead to increased toxicity.

Among the two approaches investigated, the MU‐based EL strategy allowed for more conformal and homogeneous CTV coverage across the cohort, with the cohort's mean D_95%_ in the CTV increasing to approximately 90%.On the other hand, the Central EL method, by concentrating Bragg peaks in the tumor center, resulted in a more favorable LET_d_ distribution, increasing both the minimum and maximum LET_d_. Additionally, both SHArc approaches reduced the maximum LET_d_ in the GI tract.

Plan robustness was evaluated through separate simulations accounting for ± 1.5% range uncertainty and setup errors of up to 5 mm in three Cartesian coordinates x‐y‐z (totaling 16 scenarios). Results showed that while static SHArc plans generally yielded improved minimum GTV coverage under positional uncertainties, they were more sensitive to density variations. In particular, the Central EL approach showed larger fluctuations in GTV D_95%_ and D_1%_, with more extreme minimum and maximum doses in the target under density perturbations, indicating reduced robustness. In contrast, the MU‐based EL strategy demonstrated more consistent dose metrics with less variation, indicating that the MU‐based EL method may be a more robust option for EL selection in static SHArc. In terms of GI tract dose, robustness evaluation of static SHArc revealed higher D_1cc_ metrics, reaching nearly 48 Gy(RBE) under the considered bounds of density uncertainty. However, compared to the nominal plan, 2‐SFO showed the largest increase in median change in D_1cc_ (∆D_1cc_), increasing by ∼1.8 Gy(RBE) considering both positional and density uncertainties, while ∆D_1cc_ increased by < 1 Gy(RBE) for both static SHArc plans, as shown in Figure 3. Additionally, it should be noted that the use of different α/β ratios in pancreatic cases (5 Gy for the target and 2 Gy for normal tissue) amplifies biological dose variations in surrounding organs due to an increased RBE for lower α/β tissues.

Inter‐fractional evaluations on control CTs showed that SHArc plans were highly sensitive to anatomical variations, particularly from GI tract filling and changes in the entrance channel. Unlike the 2‐SFO plans, which consider stable posterior beams irradiated through the table, SHArc's upper beams were more affected by anatomical changes. It should be noted, however, that this assessment was based on only two available control CTs and may not fully capture the cumulative effect over the entire treatment course, where dose variations could average out and lead to higher target coverage. In terms of LET_d_ distribution, it was observed that despite variations in LET_d98%_ in the GTV, these values remained higher in SHArc plans than for the 2‐beam plans. To improve inter‐fractional robustness, motion mitigation techniques such as body thermoplastic masks or corsets immobilization could be explored.^27^ However, patient comfort and the extended treatment time for SHArc must be considered. Additionally, SHArc's increased number of beams requires mitigation strategies that avoid blocking the beam path. Prone positioning could also be beneficial, as studies have shown that this position could reduce pancreatic motion in the cranio‐caudal direction.^28^

Alternatively, daily imaging and potential plan adaptation could be crucial for SHArc's clinical implementation. As an example, work by Ogawa et al. demonstrated the potential of using cone‐beam computed tomography (CBCT) for online adaptation of volumetric‐modulated arc therapy (VMAT) plans, reducing dose to the stomach and duodenum while maintaining target coverage.^29^ Similarly, MRI‐guided adaptive radiotherapy offers benefits, such as position control and real‐time plan adjustments based on synthetic CT generation, without additional radiation exposure. However, current workflow limitations, particularly treatment time, make this impractical for routine use, underlining the need for further research. AI‐based auto‐segmentation tools, such as the one presented by Shojaei^30^ could play a key role in streamlining online plan adaptation. Additionally, evaluating SHArc's inter‐fractional variation in other clinical scenarios, like brain tumors, could provide valuable insights and potentially expand SHArc's clinical applications, as these are less susceptible to anatomical changes, and may still benefit from LET_d_ boosting.

Increasing the minimum LET_d_ in larger tumors was more challenging without compromising dose distribution, leading to variations in the minimum LET_d_ objectives across plans (ranging from 50 to 80 keV/µm). For this study, a reference minimum LET_d_ of 44 keV/µm in the GTV was chosen based on previous clinical studies linking this threshold to improved local control. However, while highly relevant, these findings were derived from an 18‐patient cohort. In this line, models like the Hypoxia Reduction Factor (HRF), which incorporate oxygen and LET_d_ distribution to predict the hypoxic effective dose, could improve the assessment of LET_d_‐boosting strategies.^12^ By integrating LET_d_, these models could help evaluate whether an LET_d_ increase, and the resulting enhancement in oxygen‐sensitive dose effectiveness, can compensate for reduced plan robustness. For this, further refinement is needed to account for different cell lines and better reflect clinical conditions, particularly for pancreatic cancer.

Additionally, the difficulty in increasing LET_d_ for larger tumors while maintaining standard clinical dose constraints may suggest that selectively boosting LET_d_ in hypoxic subregions could be a more practical approach. An alternative strategy could involve relaxing dose constraints to prioritized, as explored in approaches like LEOPARD, which applies a dose boost to a hypoxic subvolume and therefore allows for a stronger LETd enhancement.^31^ However, accurately identifying these regions remains difficult due to fluctuating hypoxia patterns. Although ^18^F‐FMISO PET has been explored for hypoxia mapping in other anatomical sites, studies have shown that uptake patterns can vary over time, which raises concerns about the feasibility of consistently targeting hypoxic regions based on static imaging.^32^ Therefore, while targeting hypoxic subvolumes is promising, further research is needed to determine the most stable metric over time to guide adaptive LET_d_ escalation.

To evaluate the current possibility of delivering a SHArc plan at the heavy‐ion gantry using a step‐and‐shoot approach, a plan was optimized and irradiated, using gantry beams currently commissioned at HIT for treatment with carbon ions. Measurements at four points revealed that the largest deviation from the treatment planning system (TPS) occurred at the central point (P1), yet remained within 3%, ensuring clinical acceptability. Although we used the same cylindrical phantom as in previous SHArc dosimetric verifications, it was positioned vertically on the treatment couch for gantry‐based irradiation, introducing a potential source of deviation. Furthermore, beam path uncertainties may have increased due to interactions with the treatment couch. Unlike previous work, which used 180 beams, the current plan employed fewer beams, potentially affecting robustness.

To further investigate these deviations, we analyzed the impact of density variations in the irradiated plan, detailed in Supplementary Material (G). A simulated −1% density shift reduced the mean deviations to within 1% across all points, supporting our findings that increased beam angles and plan complexity affect robustness, a challenge further amplified by LET_d_ optimization. Both the in‐silico analysis and experimental validation underscore the need for robust optimization to account for setup and density uncertainties, as previously reported in the literature.^33^ Additionally, reducing density uncertainties may be crucial for the clinical implementation of SHArc. One potential approach is the integration of sequential dual‐energy computed tomography, which has been shown to improve range accuracy by reducing errors associated with the generalized conversion of CT numbers to stopping power ratios in single‐energy CT.^34^, ^35^, ^36^

It is widely recognized that heavy arc therapy comes as a promising strategy for LETd boosting, as the increased number of beam angles offers additional degrees of freedom to enhance LET_d_ in the GTV. However, with this work, we have also shown that heavy arc delivery alone does not guarantee a significant LET_d_ increase. The EL selection, which remains essential in static SHArc to reduce irradiation time, also has a major impact on the resulting LET_d_ distribution. Specifically, with the MU‐based EL strategy, we observed a more homogeneous but less pronounced LET_d_ boost. In such cases, one could argue that a well‐designed three‐beam configuration might achieve comparable LET_d_ levels, as we have previously investigated.^24^ However, at our institution, a three‐field setup is not considered viable for clinical implementation, since the required anterior beam would intersect the gastrointestinal tract, which is both radiosensitive and highly variable. Static SHArc, on the other hand, by spreading dose contributions over multiple beam directions, could help reduce the burden on any single path and may offer a more practical alternative. Furthermore, the use of many beam angles also helps smooth the transition between regions with different α/β ratios (i.e., tumor/normal tissue), potentially improving overall biological dose conformity.

For SHArc and other LET_d_‐boosting strategies to move towards clinical translation, future studies should incorporate LET_d_ measurements into patient‐specific quality assurance to improve treatment accuracy, similar to the approach used by Koto et al. for a head and neck patients clinical trial^37^ in which silicon‐based microdosimeters were used. For measuring higher LET_d_ values (up to 100 keV/µm), diamond detectors, as investigated by Magrin et al.^38^ could offer a more effective solution for clinical carbon ion beam dosimetry and motivate further exploration.

Finally, irradiating the SHArc plans optimized in the in‐silico analysis could take up to 28 min, for 20 beams and 140 ELs using HIT's current gantry settings. Therefore, for clinical implementation, incorporating MEM, which enables the use of multiple isoenergies within the same beam spill^39^, ^40^ will be essential to reduce treatment time, thus facilitating the translation of SHArc into clinical practice.

## 5 CONCLUSION

This study is a first step towards the clinical translation of static SHArc therapy with carbon ions for pancreatic cancer. While static SHArc can improve LET_d_ distribution within the tumor, challenges remain, particularly in optimizing plan robustness against inter‐fraction variations due to anatomical changes like filling of the GI tract. These findings also underscore the critical role of EL selection. From the two SHArc optimization strategies explored, MU‐based EL seems to be more robust and allows for improved tumor coverage, while Central EL enables further LET_d_ escalation, particularly at the tumor center. This study also motivates the potential study of SHArc for other clinical sites. Future efforts will focus on enhancing inter‐fraction robustness through motion mitigation strategies, daily imaging, and potential plan adaptation, aiming to enhance clinical applicability.

## CONFLICT OF INTEREST STATEMENT

J.L. reports receiving travel reimbursement from RaySearch Laboratories AB and Micropos Medical, speaker fee form Accuray Incorporated, outside the submitted work. J.D. reports grants from CRI The Clinical Research Institute, grants from View Ray Incl., grants from Accuray International, grants from Accuray Incorporated, grants from RaySearch Laboratories AB, grants from Vision RT limited, grants from Merck Serono GmbH, grants from Astellas Pharma GmbH, grants from AstraZeneca GmbH, grants from Siemens Healthcare GmbH, grants from Solution Akademie GmbH, grants from Eromed PLC Surrey Research Park, grants from Quintiles GmbH, grants from Pharmaceutical Research Associates GmbH, grants from Boehringer Ingelheim Pharma GmbH Co, grants from PTW‐Frieburg Dr. Pychlau GmbH, grants from Nanobiotix A.a., outside the submitted work. S.M. is supported by the Japan Society for the Promotion of Science (JSPS) fellowship for international scientists, outside the submitted work. The other authors have no relevant conflicts of interest to disclose.

## DECLARATION OF GENERATIVE AI IN SCIENTIFIC WRITING

During the preparation of this work, the author(s) used ChatGPT‐3.5 (OpenAI) in order to find synonyms/antonyms to specific words, check spelling, and explore alternative phrasing of previously written sentences to improve article readability. After using this tool/service, the author(s) reviewed and edited the content as needed and take(s) full responsibility for the content of the publication.

## Supporting information



Supporting Information
