# Supplementary material for: Development and dosimetric verification of static SHArc: Step‐and‐shoot carbon ion arc therapy for LETd escalation in pancreatic tumors
Source: Med Phys. 2025 Oct 8;52(10):e70055. doi: 10.1002/mp.70055 (PMC12505198; doi:10.1002/mp.70055)
Supplement: Supplementary file 1 — Supporting Information [file MP-52-0-s001.pdf]

Development and dosimetric verification of static SHArc: step-and-shoot carbon ion arc therapy for LET_d escalation in pancreatic tumors

Supplementary Material

<u>A: Treatment planning strategies</u>

Static SHArc plans were generated using the 20 clinically available beam angles for carbon ions at the Heidelberg Ion-beam Therapy center (HIT) illustrated in Figure A1(a). All plans were optimized following the clinical objectives used pancreas cases' plan optimization at HIT, as summarized in Table A1. Additionally, LETd optimization feature in RayStation was used for plan optimization, setting minimum LETd objectives of 50 to 80 keV/µm, based on GTV size.

As described in the Main Text, two Energy Layer (EL) selection strategies were investigated to reduce the total number of ELs in static SHArc plans for each patient. Figure A1(b) shows how these strategies lead to different EL selections for each beam, compared to the 2-SFO plan, in a representative patient case.

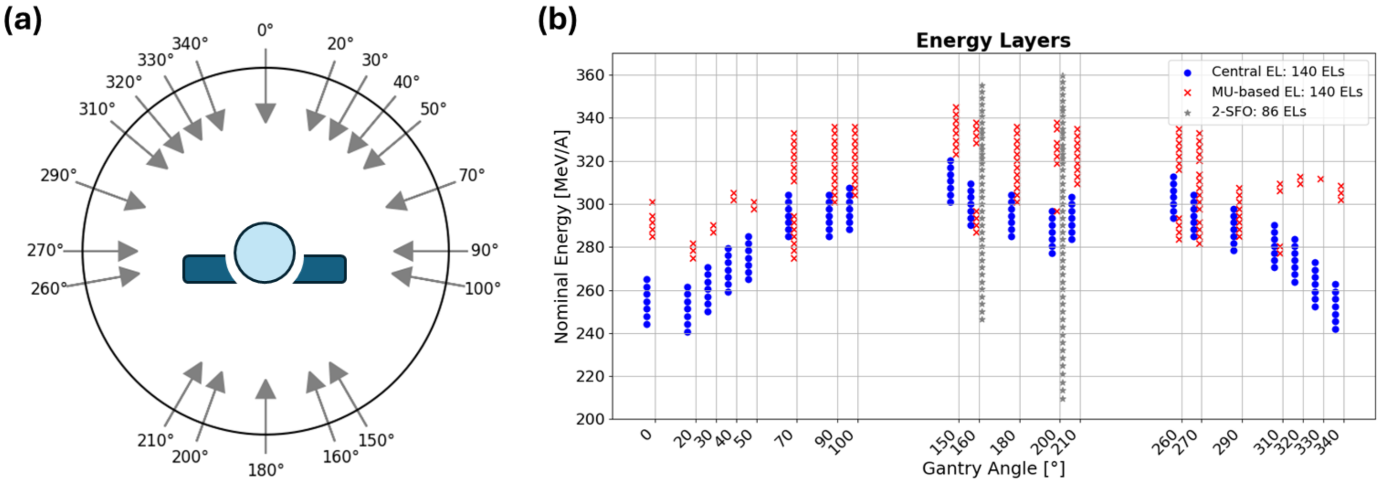

Figure A1: (a) Clinical angles available at HIT for carbon ion treatment which were used for treatment planning; (b) energy layer (EL) selection for a static SHArc optimization for a single patient case using two filtering strategies (Central EL in blue, MU-based EL in red) compared to the 2-SFO plan (gray).

Table A1: Summary of dose objectives for plan optimization. Organ at risk (OAR) constraints followed PACK trial standards, with focus on the maximum dose at the gastrointestinal tract (GI tract): including and excluding internal target volume (ITV) overlap, GI tract and GI tract-ITV respectively. Robust optimization targeted gross tumor volume (GTV) dose limits and GI tract maximum dose, considering ±7 mm setup shifts across 15 scenarios. LETd optimization aimed for a minimum of 50-80 keV/μm in the GTV.

**Treatment scheme**	48 Gy(RBE) / 12 Fx
**Target objectives**	
CTV	$D_{90\%}$ > 90% Prescribed Dose
**OAR constraints**	
GI tract	D_{max} < 45.6 Gy(RBE)
GI tract-ITV	D_{max} < 43.2 Gy(RBE)
Spinal Cord	D_{max} < 36 Gy(RBE)
Kidney	$V_{24\ Gy(RBE)} < 20\%$
**Goals for robust optimization**	
GTV	D_{min} > 44 Gy(RBE)
GTV	D_{max} < 50.5 Gy(RBE)
GI tract	D_{max} < 43.2 Gy(RBE)
**Goals for LET_d optimization**	
GTV	Min. LET_d 50-80 kev/μm
CTV	Min. LET_d 40 kev/μm

B: NTCP evaluations

Normal Tissue Complication Probability (NTPC) for different toxicity endpoints in the GI tract was evaluated based on Lyman-Kutcher-Burman (LKB). For that, the biologically equivalent doses in 2-Gy fractions (EQD_2) were calculated using an alpha/beta ratio of 4 Gy, specific to GI tract toxicity[S1,S2]. The LKB model requires three parameters: TD50 (the tolerance dose for 50% probability of toxicity to an organ), m (which defines the steepness of the dose-response curve), and n (which accounts for the organ's volume effect). These parameters were obtained from published studies to estimate GI tract toxicity risks across different planning strategies, as shown in Table B1, and used to calculate the NTCP for different endpoints.

Table B1: Parameters (TD50, m and n) considered for the evaluation of the Normal Tissue Complication Probability (NTCP) at the gastrointestinal tract (GI tract) using the Lyman-Kutcher-Burman model for different clinical endpoints.

GI tract: clinical endpoint	TD50 [Gy(RBE)]	m	n	Reference
Gastrointestinal hemorrhage	52.5	0.35	0.21	(Burman et al., 1991)
Obstruction/Perforation	55	0.16	0.15	(Lyman, 1985)
Diarrhea (Intestine)	55	0.79	0.15	(Reinartz et al., 2021)

Figure B1 shows NTCP comparisons for the different endpoints evaluated, for the three planning approaches. The NTCP calculations for three different endpoints revealed no significant differences between the static SHArc approaches and 2-SFO, neither in terms of improvement nor deterioration. The largest difference in mean NTCP was observed for the diarrhea endpoint, in which the mean NTCP increased 0.406% and 0.367% for the Central EL and MU-based EL strategies, respectively, in comparison to the 2-SFO plan. This may also indicate that the increase in the low-dose bath observed in the GI tract for the SHArc plans does not have a meaningful impact on NTCP predictions.

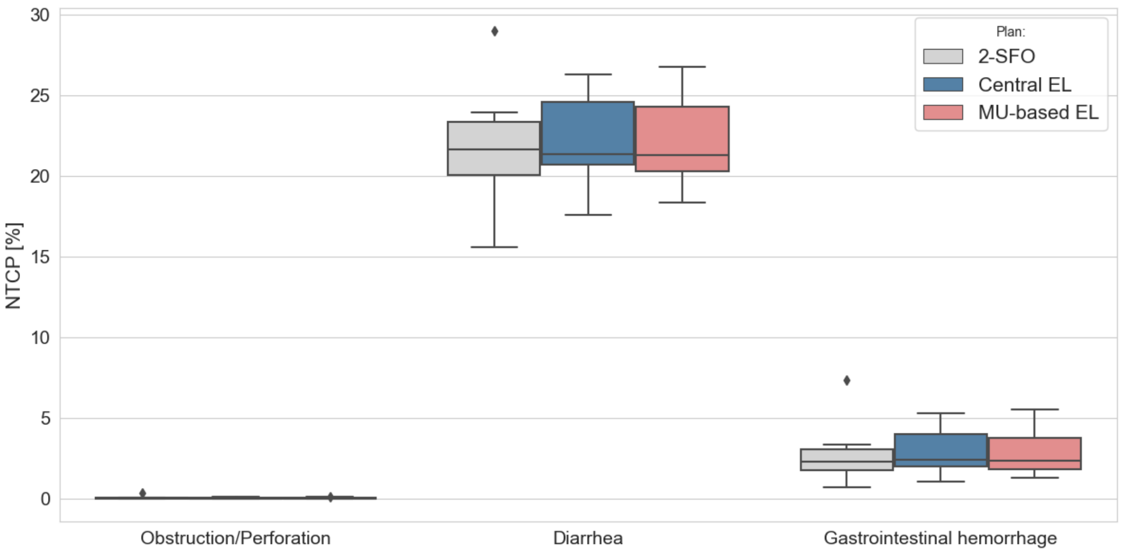

Figure B1: NTCP comparison for different clinical endpoints across the three planning strategies: 2-SFO (gray), Central EL (blue), and MU-based EL (red). The NTCP values were calculated using the parameters shown in table B1, and considering a α/β=4 Gy to compute the EQD_2.

C: Statistical analysis

Statistical analysis of the dose metrics presented in Table 1 (in the main manuscript) and of additional LET_d metrics relevant to this study was performed to compare the different planning approaches using pair-wise two-sided Wilcoxon signed-rank tests. A p-value below 0.05 was considered statistically significant. All analyses were performed using SciPy. The small sample size of seven patients should be considered when interpreting the results.

Table C1: Statistical analysis of **target's dose coverage**. Pairwise comparisons of target dose metrics between planning strategies (2-SFO, Central EL, MU-based EL). Values shown are mean differences between plans, 95% confidence intervals (CI), and associated p-values from two-sided Wilcoxon signed-rank tests. Statistically significant results ($p < 0.05$) are highlighted. The mean difference is calculated as: mean of the second-listed strategy minus the first.

Target: dose coverage	Plan comparison	Mean Difference	95% CI	p-value
CTV: $D_{95\%}$ [Gy(RBE)]	2-SFO vs Central EL	1.43	[-0.09, 2.97]	0.156
	2-SFO vs MU-based EL	2.43	[1.01, 3.98]	**<0.05**
	Central EL vs MU-based EL	1.00	[0.78, 1.21]	**<0.05**
CTV: $D_{90\%}$ [Gy(RBE)]	2-SFO vs Central EL	1.00	[-0.27, 2.52]	0.219
	2-SFO vs MU-based EL	2.07	[0.82, 3.62]	**<0.05**
	Central EL vs MU-based EL	1.06	[0.74, 1.32]	**<0.05**
CTV: $V_{95\%Dpres}$ [%]	2-SFO vs Central EL	-0.82	[-6.06, 3.55]	0.938
	2-SFO vs MU-based EL	7.75	[4.2, 10.18]	**<0.05**
	Central EL vs MU-based EL	8.56	[5.49, 11.4]	0.016
CTV: HI	2-SFO vs Central EL	-0.04	[-0.07, 0.0]	0.219
	2-SFO vs MU-based EL	-0.05	[-0.09, -0.01]	0.078
	Central EL vs MU-based EL	-0.02	[-0.02, -0.02]	**<0.05**

Table C1 (continued): Statistical analysis of **OAR dose metrics and LETd metrics for GTV and gastrointestinal tract (GI tract)**. Pairwise comparisons of target dose metrics between planning strategies (2-SFO, Central EL, MU-based EL). Values shown are mean differences between plans, 95% confidence intervals (CI), and associated p-values from two-sided Wilcoxon signed-rank tests. Statistically significant results ($p < 0.05$) are highlighted. The mean difference is calculated as: mean of the second-listed strategy minus the first.

OAR: dose coverage	Plan comparison	Mean Difference	95% CI	p-value
GI tract: D_{max} [Gy(RBE)]	2-SFO vs Central EL	0.93	[-1.11, 2.79]	0.578
	2-SFO vs MU-based EL	1.14	[-1.17, 3.12]	0.297
	Central EL vs MU-based EL	0.21	[-0.07, 0.57]	0.375
GI tract-ITV: D_{max} [Gy(RBE)]	2-SFO vs Central EL	0.19	[-1.33, 1.68]	0.813
	2-SFO vs MU-based EL	0.15	[-1.44, 1.57]	0.938
	Central EL vs MU-based EL	-0.04	[-0.12, 0.02]	0.469
Spinal Cord D_{max} [Gy(RBE)]	2-SFO vs Central EL	-14.99	[-17.84, -12.33]	**<0.05**
	2-SFO vs MU-based EL	-13.23	[-16.21, -10.41]	**<0.05**
	Central EL vs MU-based EL	1.77	[-2.4, 5.36]	0.375
Kidney (right): $V_{24Gy(RBE)}$ [%]	2-SFO vs Central EL	-2.88	[-7.53, 1.1]	0.345
	2-SFO vs MU-based EL	-3.05	[-8.54, 1.82]	0.345
	Central EL vs MU-based EL	-0.17	[-1.41, 1.05]	0.854
Kidney (left): $V_{24Gy(RBE)}$ [%]	2-SFO vs Central EL	-4.21	[-7.65, -0.8]	0.078
	2-SFO vs MU-based EL	-3.72	[-7.36, 0.17]	0.109
	Central EL vs MU-based EL	0.49	[-0.03, 1.34]	0.285
Body: $V_{4.8Gy(RBE)}$ [%]	2-SFO vs Central EL	11.61	[6.33, 19.67]	**<0.05**
	2-SFO vs MU-based EL	11.12	[5.77, 18.89]	**<0.05**
	Central EL vs MU-based EL	-0.49	[-1.12, 0.21]	0.297

Target: LET_d coverage	Plan comparison	Mean Difference	95% CI	p-value
GTV: $LET_{d98\%}$ [keV/µm]	2-SFO vs Central EL	18.39	[12.7, 25.1]	**<0.05**
	2-SFO vs MU-based EL	16.53	[11.61, 21.72]	**<0.05**
	Central EL vs MU-based EL	-1.86	[-4.5, 0.63]	0.297
GTV: $LET_{d1\%}$ [keV/µm]	2-SFO vs Central EL	37.77	[32.06, 44.13]	**<0.05**
	2-SFO vs MU-based EL	-3.30	[-11.06, 6.01]	0.578
	Central EL vs MU-based EL	-41.07	[-46.1, -32.84]	**<0.05**
GI tract: $LET_{d1\%}$ [keV/µm]	2-SFO vs Central EL	-38.44	[-46.49, -25.36]	**<0.05**
	2-SFO vs MU-based EL	-29.69	[-36.82, -21.24]	**<0.05**
	Central EL vs MU-based EL	8.76	[0.17, 15.69]	0.109

D: Patient specific DVH and LVH

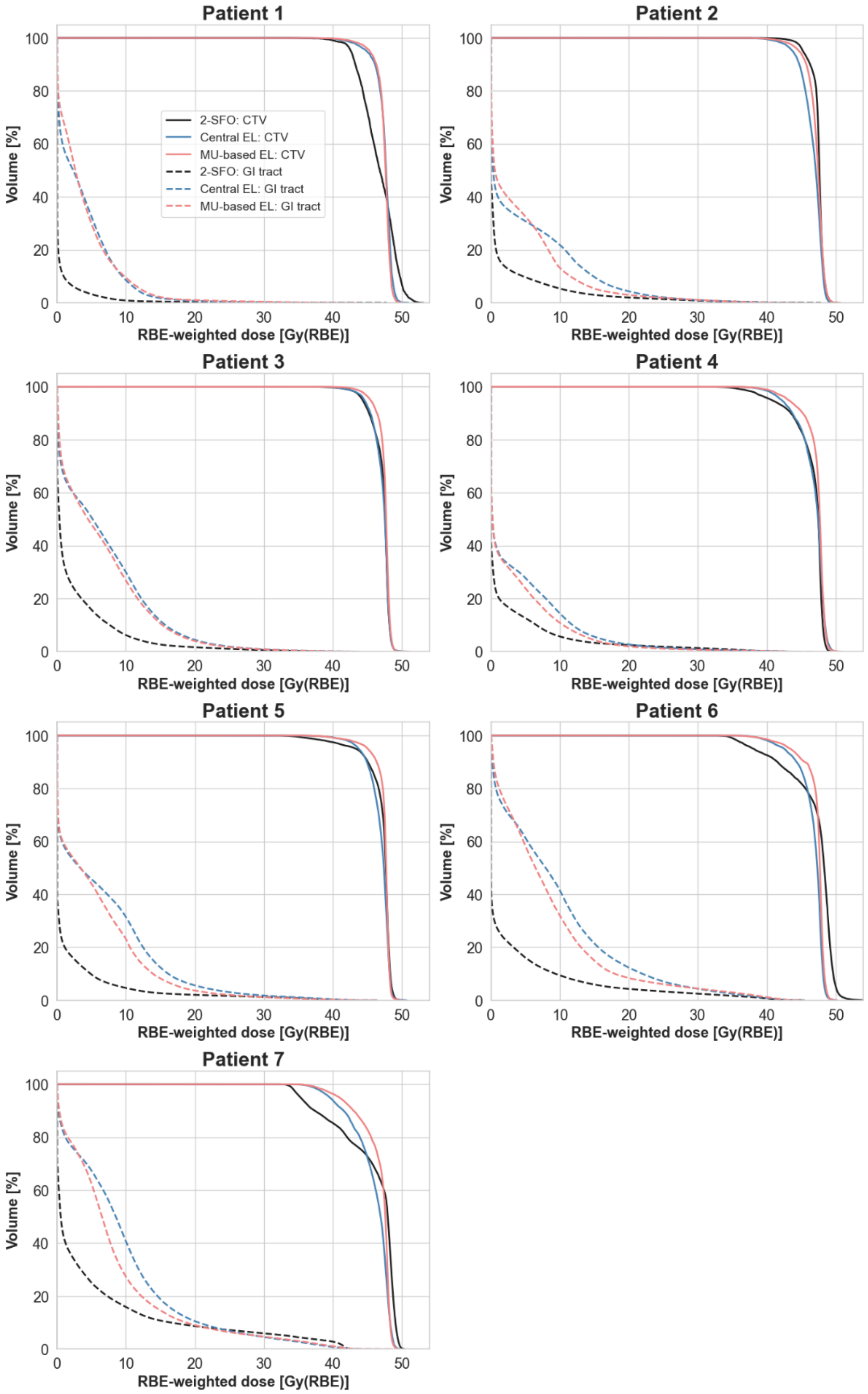

Figure D1: Dose-volume histogram for each patient, showing the RBE-weighted dose in the clinical target volume (CTV) (solid line) and the gastrointestinal tract (GI tract) (dashed line) for each planning strategy: 2-SFO (black), SHArc – Central EL (red), and SHArc – MU-based EL (blue).

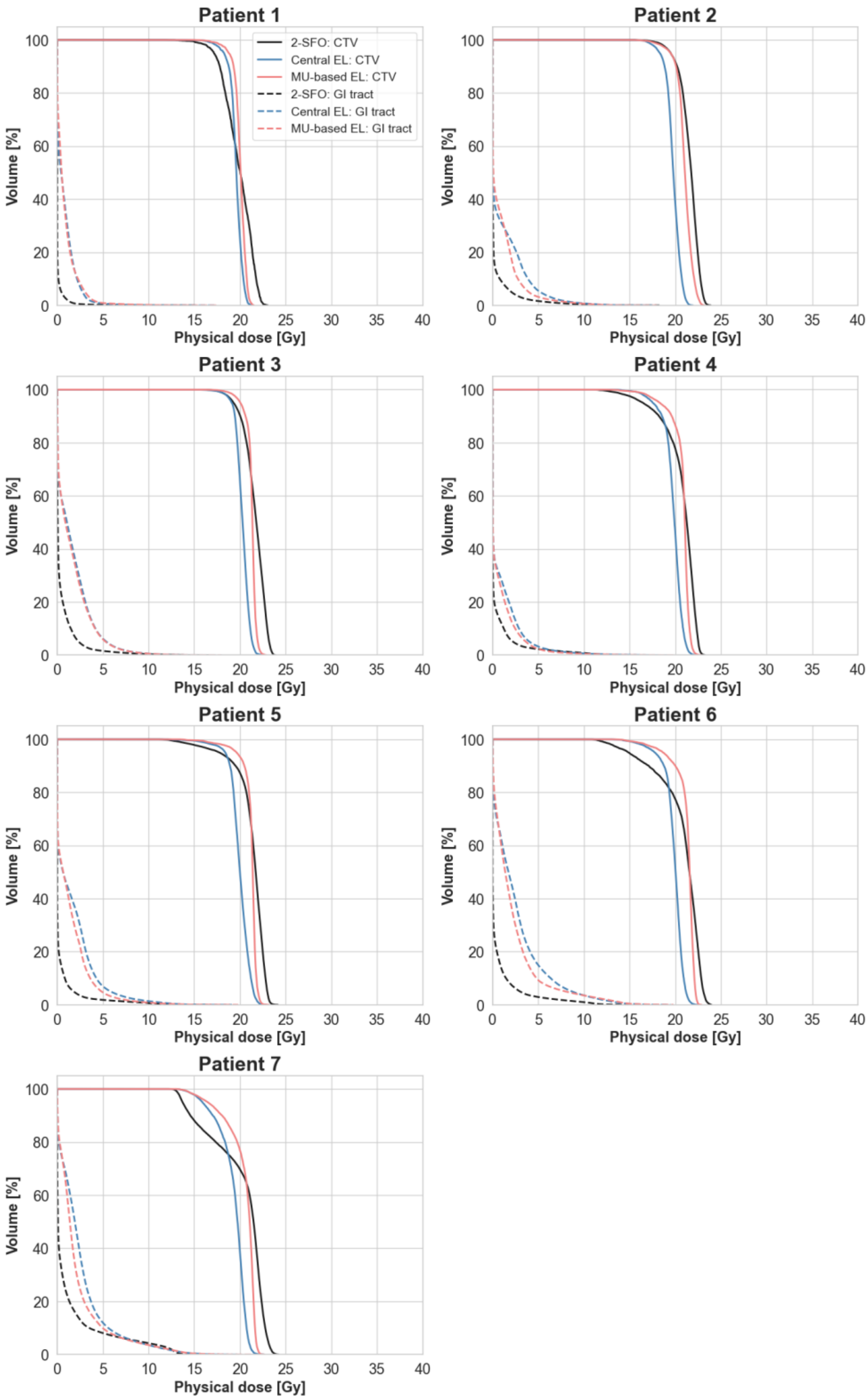

Figure D2: Dose-volume histogram for each patient, showing the Physical dose in the clinical target volume (CTV) (solid line) and the gastrointestinal tract (GI tract) (dashed line) for each planning strategy: 2-SFO (black), SHArc – Central EL (red), and SHArc – MU-based EL (blue).

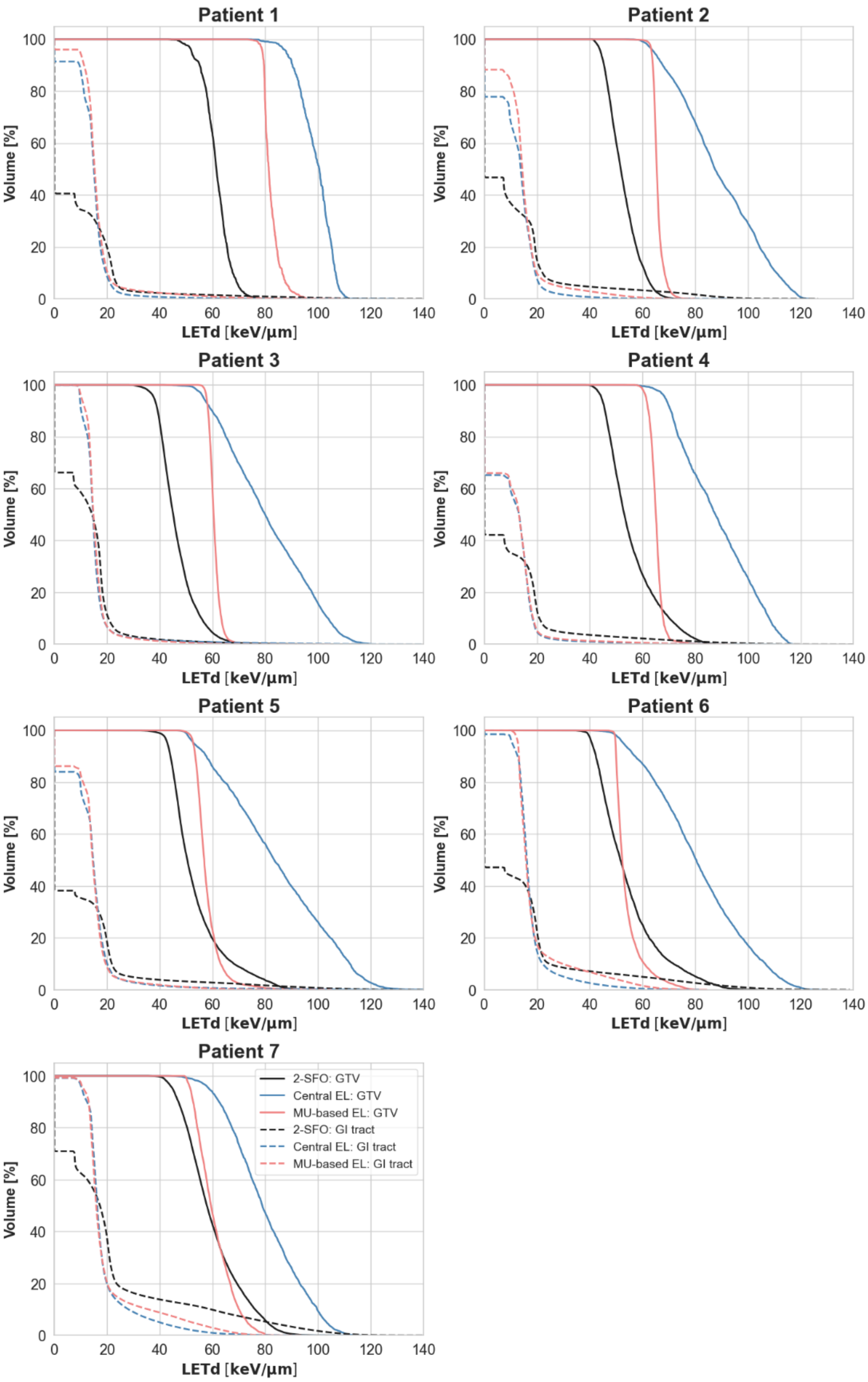

Figure D3: Dose-volume histogram for each patient, showing the LETd in the clinical target volume (CTV) (solid line) and the gastrointestinal tract (GI tract) (dashed line) for each planning strategy: 2-SFO (black), SHArc – Central EL (red), and SHArc – MU-based EL (blue).

E: LET_d distribution for a patient case

Figure E1 presents axial slices of the LET_d distribution for one patient for the 2-SFO, Central EL, and MU-based EL plans, as well as the corresponding LET_d profiles, extracted along the white line. The figure highlights the high LET_d region in the tumor center achieved with the Central EL strategy, in contrast to the more uniform distribution observed with the MU-based EL approach.

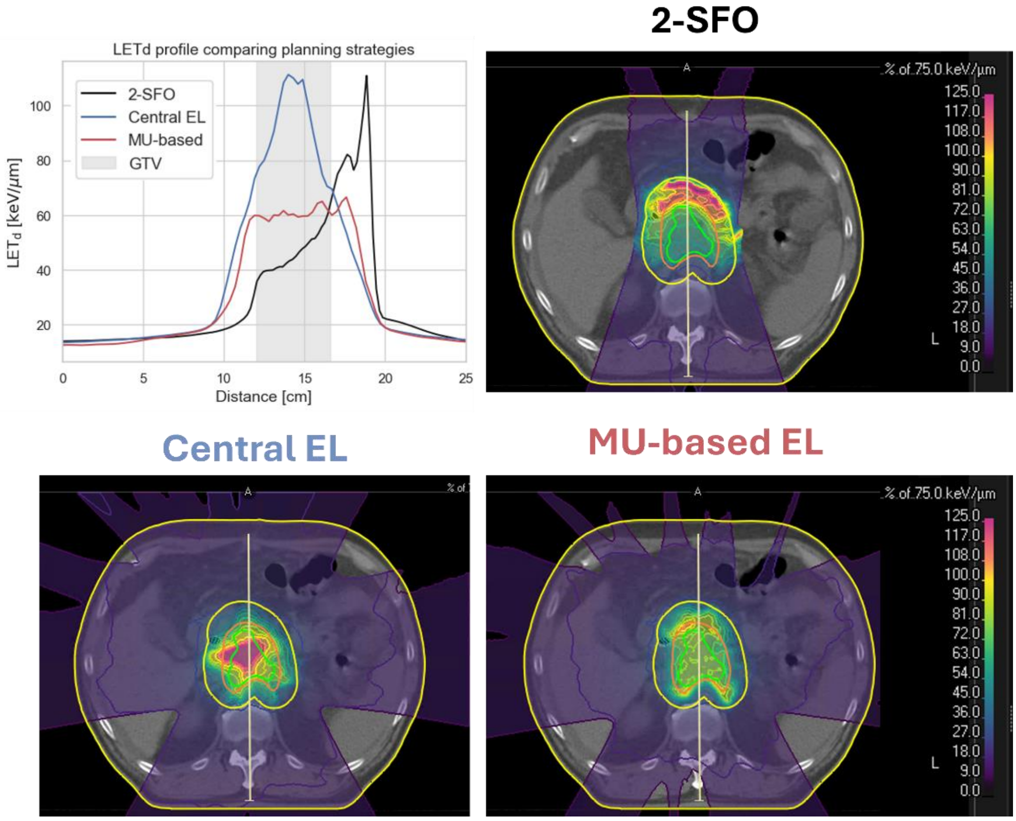

Figure E1: Axial slice of LET_d distribution for a single patient across the three optimization strategies studied: 2-SFO, static SHArc: Central EL and static SHArc: MU-based EL. The LET_d distribution is shown for voxels with a minimum dose threshold of least 10% of the prescribed dose. The target is shown in orange (clinical target volume) and green (gross tumor volume). Additionally, a comparison of the respective LET_d profiles across a line (in white) is represented as a function of penetrated depth (in cm).

F: Inter-fractional variation for a patient case

A static SHArc plan using Central EL is shown below, demonstrating significant tumor coverage loss between the planning CT and control CT 1, highlighting the impact of anatomical changes on dose distribution.

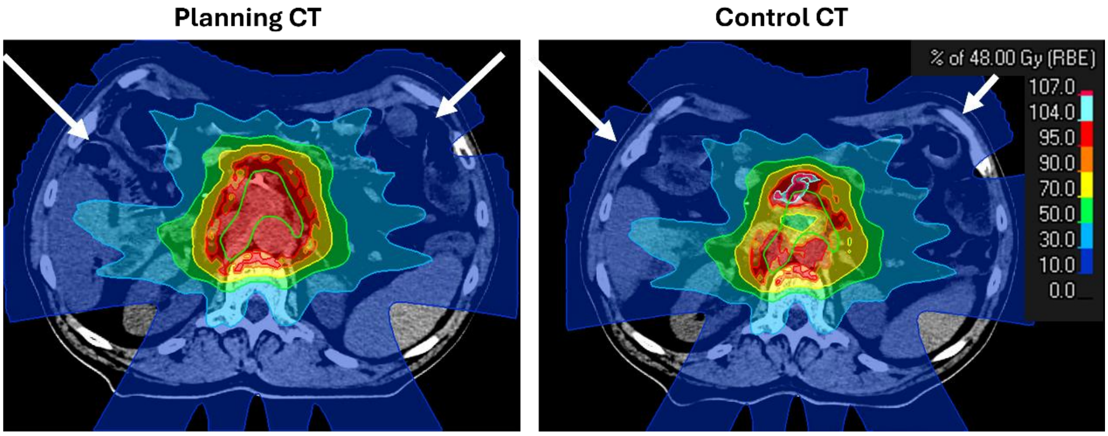

Figure F1: RBE-weighted dose distribution for a patient case where $V_{95\%}$ in the gross tumor volume (GTV) decreases from approximately 97% in the initial plan, to 25% in control CT following forward dose recalculation. White arrows indicate regions with anatomical variation, such as variations in GI tract filling. The GTV is shown in green.

G: Dosimetric verification at the HIT gantry treatment room

The static SHArc plan optimized for delivery at the heavy-ion gantry using a cylindrical PMMA phantom followed a step-and-shoot approach, ensuring a uniform and conformal 1 Gy physical dose to the target. This optimization aimed to enhance robustness against positional uncertainties in the experimental setting. Consistent with the in-silico analysis, the plan incorporated 20 clinically commissioned beam angles at HIT.

Although measurement deviations from the TPS remained below 3%, several factors, particularly density uncertainty, may have contributed to these discrepancies. To investigate this, we performed forward calculations of the planned dose under ±1% density perturbations. Figure G1 illustrates the resulting dose distributions, showing that a -1% density perturbation would lead to underdosage in the central region, consistent with the experimentally observed underdosage at P1. Table G1 summarizes the deviations between expected and measured values, including those accounting for density variations.

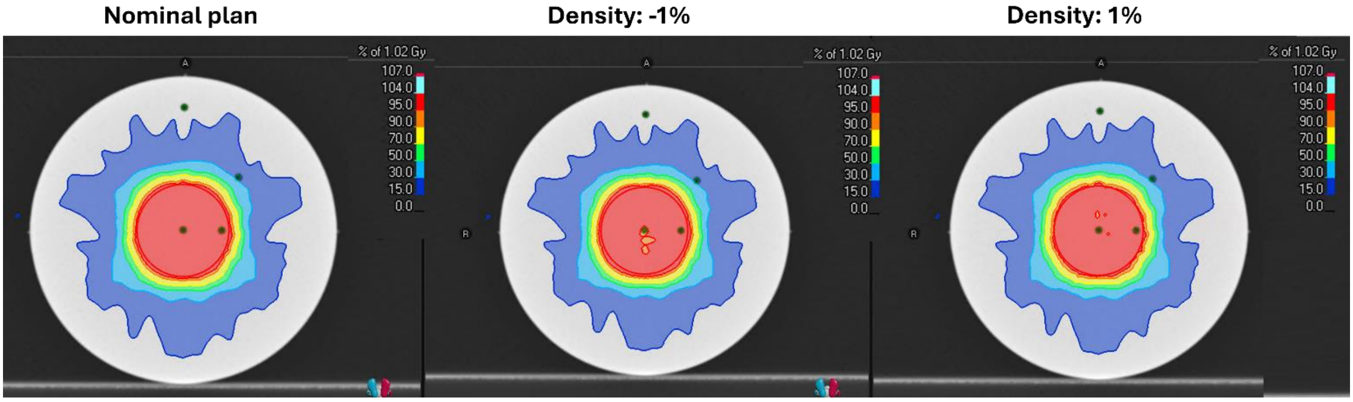

Figure G1: Axial slice of the delivered plan's physical dose distribution, with the clinical target volume (CTV) outlined in red and measurement points (P1–P4) marked in green. The left image shows the planned dose, while the middle and right images show dose distributions under -1% and +1% density perturbations, respectively.

Table G1: Deviation of dosimetric measurements from treatment planning system (TPS) calculations, where TPS values were taken from the nominal plan and ±1% density perturbation scenarios. Deviations were reported for each measurement point (P1–P4) following clinical QA procedures.

	Mean deviation to TPS (%)		
**Position**	**Nominal plan**	**Perturbed dose: -1%**	**Perturbed dose: 1%**
P1	-2.75%	-0.02%	-5.09%
P2	-0.38%	-0.69%	-0.1%
P3	+0.86%	+0.91%	+0.52%
P4	+0.22%	+0.19%	+0.25%

Additional references:

[S1] Deore SM, Viswanathan PS, Shrivastava SK, Supe SJ, Dinshaw KA. Predictive role of tdf values in late rectal recto-sigmoid complications in irradiation treatment of cervix cancer. Int J Radiat Oncol Biol Phys 1992;24:217–21.https://doi.org/10.1016/0360-3016(92)90674-7.

[S2] Dische S et al. Carcinoma of the cervix and the use of hyperbaric oxygen with radiotherapy: a report of a randomised controlled trial. Radiother Oncol Nov.1999;53:93–8. https://doi.org/10.1016/S0167-8140(99)00124-3.
